# The complete mitochondrial genome sequence of *Pelates quadrilineatus* (Perciformes: Terapontidae)

**DOI:** 10.1080/23802359.2017.1413304

**Published:** 2018-01-15

**Authors:** Wei Shi, Shixi Chen, Hui Yu

**Affiliations:** a College of Life Science, Foshan University, Foshan, Guangdong, China;; b CAS Key Laboratory of Tropical Marine Bio-resources and Ecology, South China Sea Institute of Oceanology, Chinese Academy of Sciences, Guangzhou, China

**Keywords:** Perciformes, *Pelates quadrilineatus*, phylogenetic relationship

## Abstract

The complete mitochondrial genome of marine fish *Pelates quadrilineatus* was sequenced by the high throughput sequencing method. This genome was 16,823 bp in length, consisted of 13 protein-coding genes, 22 tRNA genes, two rRNA genes and one large non-coding region. The gene arrangement of *P. quadrilineatus* is identical to those of other fishes. Phylogenetic tree based on 13 protein coding genes shows that Terapontidae has a closer phylogenetic relationship to Pentacerotidae than to Chaetodontidae.

*Pelates quadrilineatus*, common name Fourlined Terapon, inhabits in brackish waters (Kuiter and Tonozuka 2001) and feeds on small fishes and invertebrates. Eggs of this species are hatched by the male parent (Breder and Rosen 1966), juveniles usually live in seagrass beds or mangrove bays (Kuiter and Tonozuka 2001). Now *P*. *quadrilineatus* has been classified into family Terapontidae, order Perciformes (Nelson et al. 2016), because the morphological differences between Terapontidae fishes are not obvious. The fringes of body side, which is main identified character of *P*. *quadrilineatus*, change greatly at different stages of maturity (Vari 1978). This phenomenon has made it necessary to develop molecular markers to figure out controversial issues about phylogenetic relationship of *P*. *quadrilineatus* (Paxton 1989; Faith et al. 2004). In this study, we first reported the complete mitochondrial genome of *P*. *quadrilineatus*, and analyzed its phylogenetic relationship with some other species from family Pentacerotidae and Chaetodontidae, based on samples collected from Naozhou island in Zhanjiang, China (geographic coordinate: N 20°53′20.11″, E 112°28′46.2″). The whole body specimen was preserved in ethanol and registered to the Marine Biodiversity Collection of South China Sea, Chinese Academy of Sciences, under the voucher number SCF20171022001.

The complete mitochondrial genome of *P*. *quadrilineatus* was 16,823 bp in length (GenBank accession no. MG271911), including 13 protein-coding genes, two rRNA genes, 22 tRNA genes, one OL (origin of replication on the light-strand) and one D-Loop (control region). The OL was 51 bp in length, located in the cluster of five tRNA genes (*WANCY* region) between *tRNA*-*Asn* and *tRNA*-*Cys*. The D-loop was 956 bp in length, located between *tRNA*-*Pro* and *tRNA*-*Phe*. Gene arrangement of this genome was identical to those of other fish, and most genes in this genome were encoded by the heavy strand (H-strand), except for *ND6* and eight tRNA genes (Boore 1999; Yu and Kwak 2015; Gong et al. 2017). Overall base composition values for the mitochondrial genome were 27.3%, 30.2%, 16.7%, and 25.8% for A, C, G, and T, respectively.

The phylogenetic relationships of *P*. *quadrilineatus* with 11 closely related species were analyzed in this study. Complete mitochondrial genes of these 11 species were available on GenBank. The maximum-likelihood evolutionary tree (ML tree) was constructed by MEGA 7 (Kumar et al. 2016) based on 1st and 2nd codon sequences of 13 protein coding genes.

In the ML phylogenetic tree, *P*. *quadrilineatus* clustered with *Terapon jarbua*, *Rhynchopelates oxyrhynchus*, *Scortum barcoo* and *Bidyanus bidyanus* with a strong support. And these five species were all classified into family Terapontidae of order Perciformes. As a sister lineage to the former mentioned clade, *Histiopterus typus*, *Pseudopentaceros richardsoni* and *Pentaceros japonicus* formed another clade, which was classified into family Pentacerotidae of order Perciformes. Another clade included *Chaetodon auripes*, *Heniochus diphreutes and Chelmon rostratus* with a strong support, which was classified into family Chaetodontidae of order Perciformes (Figure 1). These results show that Terapontidae has a closer phylogenetic relationship to Pentacerotidae than to Chaetodontidae.

**Figure 1.**
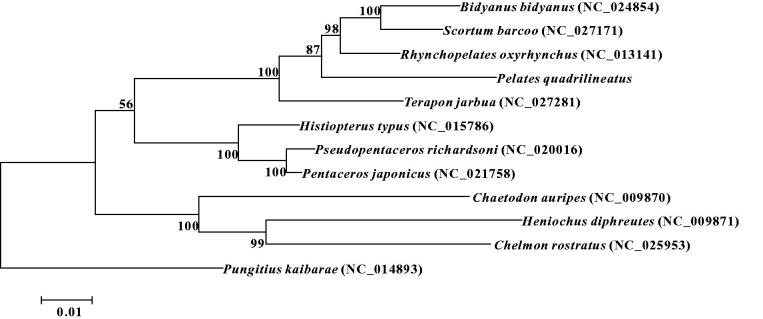
Maximum-likelihood phylogenetic tree was constructed based on 1st and 2nd codon sequences of 13 protein coding genes of 11 species.
